# Dominant spinal muscular atrophy is caused by mutations in BICD2, an important golgin protein

**DOI:** 10.3389/fnins.2015.00401

**Published:** 2015-11-05

**Authors:** Lilian A. Martinez-Carrera, Brunhilde Wirth

**Affiliations:** Institute of Human Genetics, Institute for Genetics and Center for Molecular Medicine of The University of CologneCologne, Germany

**Keywords:** BICD2, SMALED2, SMA, Golgi fragmentation, endocytosis, RAB6A, dynein, dynactin

## Abstract

Spinal muscular atrophies (SMAs) are characterized by degeneration of spinal motor neurons and muscle weakness. Autosomal recessive SMA is the most common form and is caused by homozygous deletions/mutations of the *SMN1* gene. However, families with dominant inherited SMA have been reported, for most of them the causal gene remains unknown. Recently, we and others have identified heterozygous mutations in *BICD2* as causative for autosomal dominant SMA, lower extremity-predominant, 2 (SMALED2) and hereditary spastic paraplegia (HSP). *BICD2* encodes the Bicaudal D2 protein, which is considered to be a golgin, due to its coiled-coil (CC) structure and interaction with the small GTPase RAB6A located at the Golgi apparatus. Golgins are resident proteins in the Golgi apparatus and form a matrix that helps to maintain the structure of this organelle. Golgins are also involved in the regulation of vesicle transport. *In vitro* overexpression experiments and studies of fibroblast cell lines derived from patients, showed fragmentation of the Golgi apparatus. In the current review, we will discuss possible causes for this disruption, and the consequences at cellular level, with a view to better understand the pathomechanism of this disease.

Spinal muscular atrophy (SMA) is a diverse group of genetic disorders characterized by aberrant development and/or loss of spinal motor neurons, and muscle weakness without sensory neuron involvement (Wee et al., 2010). SMA is usually classified by the pattern of weakness (i.e., proximal or distal) and mode of inheritance (autosomal recessive, autosomal dominant, X-linked).

Autosomal recessive SMA linked to chromosome 5 (5q-SMA) is the most common form accounting for up to 95% of the SMA cases, and is caused by homozygous deletion/mutation of the survival motor neuron 1 (*SMN1*) gene, localized on chromosome 5q12-q13 (Lefebvre et al., 1995; Wirth, 2000). The 5q-SMA is classified into different types based on onset and severity of symptoms. The most severe form of 5q-SMA (type I) has an onset within the first 6 months of life, permanent inability to seat without support and usually death within 2 years (Iannaccone et al., 1993; Rudnik-Schöneborn et al., 1996). The intermediate form of 5q-SMA (type II) has an onset between 6 and 18 months of life, ability to seat but not to walk and death usually occurs after the age of 2 years. A milder form is the 5q-SMA type III with an onset at around 3 years of age, ability to stand and walk but because of progressive muscle weakness, the patients mostly are wheelchair-bound as the disease progresses (Zerres and Rudnik-Schöneborn, 1995; Zerres et al., 1995). The 5q-SMA type IV, also called “adult SMA,” is considered the mildest and less severe form with a late onset of around 30 years of age (Zerres et al., 1995). All the mentioned forms of 5q-SMA commonly share proximal muscle weakness with slow progression in case of type II-IV SMA.

In contrast to autosomal recessive 5q-SMA, the autosomal dominant SMA (ADSMA) forms are less frequent, milder, with vague progression and probably, even under-diagnosed. In approx. 70% of individuals with ADSMA the genetic cause is still unknown. However, due to remarkable advances in next generation sequencing and gene discovery, the number of newly described causative genes increases continuously.

Beside autosomal dominant SMA lower extremity-predominant 1 (SMALED1, OMIM #58600) caused by mutations in dynein cytoplasmic 1 heavy chain 1 protein (*DYNC1H1*; OMIM ^*^600112), we and others have recently identified heterozygous variants in *BICD2* (OMIM ^*^609797) as causative for autosomal dominant SMA, lower extremity-predominant, 2, (SMALED2; OMIM #615520) and hereditary spastic paraplegia (HSP) (Neveling et al., 2013; Oates et al., 2013; Peeters et al., 2013).

## Clinical features of individuals carrying mutations in BICD2

Individuals carrying heterozygous missense variants in *BICD2* exhibit muscle weakness and atrophy predominantly of the proximal lower limbs. However, in some cases the distal lower limbs are also affected, and in few others upper limbs are additionally compromised. The initial reasons for clinical counseling are commonly the difficulties in walking (waddling gait and toe walking), and delayed motor milestones. Contractures are reported frequently, and few patients present congenital hip dysplasia. Notoriously, many patients show evident wasting of the lower limbs and a very broad upper body, which resembles a bodybuilder-like shape. Symptoms are usually present at birth or appear in early childhood, but some cases with adult onset have been also described (Table 1; Neveling et al., 2013; Oates et al., 2013; Peeters et al., 2013; Synofzik et al., 2014; Rossor et al., 2015). The course of disease is slowly progressive or non-progressive. Due to the mild phenotype, patients are sometimes diagnosed as SMA type IV, the mildest form of the classical 5q-SMA. However, most cases of SMALED2 present a congenital or early onset with foot deformities and joint contractures, and difficulties when began walking (Frijns et al., 1994; Adams et al., 1998; Oates et al., 2012; Neveling et al., 2013). One infant who died of other causes at 14 months of age showed in the post-mortem examination decreased number of anterior horn cells in the lumber and cervical spine with no peripheral nerve pathology confirming a SMA (Oates et al., 2012).

**Table 1:** **Clinical summary of SMALED2 patients carrying BICD2 substitutions**.

**Variant/Mutation**	**Onset**	**x/y**	**Motor ability**	**x/y**	**Muscle weakness**	**x/y**	**Muscle atrophy**	**x/y**	**Contractures**	**x/y**	**Congenital hip dysplasia. x/y**	**References**
c.320C>T p.Ser107Leu	Congenital < 3 years 3–12 years	9/29 10/29 10/29	Delayed motor milestones Waddling gait Toe walking	10/29 25/29 7/29	LL prox and distal LL prox Additional UL prox Additional UL distal	18/29 11/29 1/29 2/29	LL prox and distal LL prox Shoulder girdle	18/29 5/29 10/29	Foot Hips Ankles Achiles tendon	15/29 3/29 7/29 4/29	2/29	Neveling et al., 2013 Peeters et al., 2013 Oates et al., 2013
c.563A>C p.Asn188Thr	< 3 years	4/5	Delayed motor milestones Waddling gait Toe walking	1/5 2/5 1/5	LL prox	5/5	LL prox	5/5	no		4/5	Neveling et al., 2013
c.565A>T p.Ile189Phe	Congenital	1/1	Delayed motor milestones Wheelchair dependent	1/1 1/1	LL prox and distal Additional UL	1/1 1/1	LL prox and distal	1/1	Ankles, knees and hips	1/1	1/1	Oates et al., 2013
c.1502G>C p.Arg501Pro	Congenital 3–12 years	2/5 3/5	Delayed motor milestones Waddling gait Toe walking	2/5 3/5 3/5	LL prox and distal LL distal	2/5 2/5	LL prox and distal	4/5	Foot Achiles tendon	3/5 3/5	1/5	Oates et al., 2013
c.1523A>C p.Lys508Thr	Adulthood	1/1	Abnormal gait, difficulty walking	1/1	LL prox and distal	1/1	LL distal	1/1	Hips and knees	1/1	No	Oates et al., 2013
c.1604C>T p.Ala535Val	Congenital	2/2	Delayed motor milestones Waddling gait	2/2 2/2	LL prox and distal	2/2	LL prox and distal	2/2	Foot	2/2	1/2	Rossor et al., 2015
c.2108C>T p.Thr703Leu	Congenital	2/2	Difficulty walking Crutch-assisted	2/2 1/2	LL prox and distal Additional UL distal	2/2 2/2	LL prox	2/2	Foot	2/2	No	Neveling et al., 2013
c.2239C>T p.Arg747Cys	3–12 years Adulthood	1/3 2/3	Toe walking Difficulty walking and climbing	3/3 3/3	LL prox	3/3	LL prox	3/3	no		No	Synofzik et al., 2014
c.2321A>G p.Glu774Gly	3–12 years	1/1	Delayed motor milestones Waddling gait	1/1 1/1	LL prox and distal	1/1	LL prox and distal	1/1	no		No	Peeters et al., 2013

*x/y, x of a total of y cases present feature; LL, lower limbs; UL, upper limbs*.

Two families affected by an HSP phenotype associated to *BICD2* variants, have been described (Oates et al., 2013; Novarino et al., 2014). In the first family, the affected individuals presented features in adulthood, and showed lower-limb spasticity and hyperreflexia as is typical for HSP. It is also reported, a slow progression of contractures, weakness, and wasting (Oates et al., 2013). Regarding the association of this specific variant with the development of HSP but not SMALED2, it would be of particular interest to exclude the involvement of secondary disease-causing genes, resulting then in a multigenic disease, instead of a monogenic form. The second family with HPS linked to *BICD2* is consanguineous, and the affected individuals carry the homozygous variant in *BICD2*, c.G1823A, p.S608L (Novarino et al., 2014). This variant was identified by whole-exome sequencing in combination with network analysis, and is the only homozygous variant reported in *BICD2* so far.

Numerous experimental attempts have been made leading to a better understanding of the physiological role of BICD2 and yielded first insights into possible effects of variants in *BICD2* on a cellular and molecular level (Table 2). However, further investigation is needed to fully understand the pathomechanism of how mutations in BICD2 can cause SMALED2 and rarely HSP-like phenotype. Next, we summarize the mutation spectrum and functional knowledge about BICD2, and focus on Golgi fragmentation as a cellular consequence of certain BICD2 mutations, as a possible mechanism to explain failure in neuronal development and maintenance.

**Table 2:** **Summary of possible molecular mechanism involved in SMALED2 due to BICD2 substitutions**.

**Variant/Mutation**	**Mechanism proposed**	**Mechanism proposed by**
c.320C>T/p.Ser107Leu	Higher interaction with DIC and p150Glued subunit of dynactin	Oates et al., 2013; Peeters et al., 2013
c.563A>C/p.Asn188Thr	Slight Golgi fragmentation	Neveling et al., 2013
c.1502G>C/p.Arg501Pro	Higher interaction with DIC and p150Glued subunit of dynactin	Oates et al., 2013
c.2108C>T/p.Thr703Leu	Prominent Golgi fragmentation	Neveling et al., 2013
c.2321A>G/p.Glu774Gly	Less interaction with Rab6a	Peeters et al., 2013

## Genetic spectrum of pathogenic variants found in *BICD2* in patients with SMALED2

**Figure 1:**
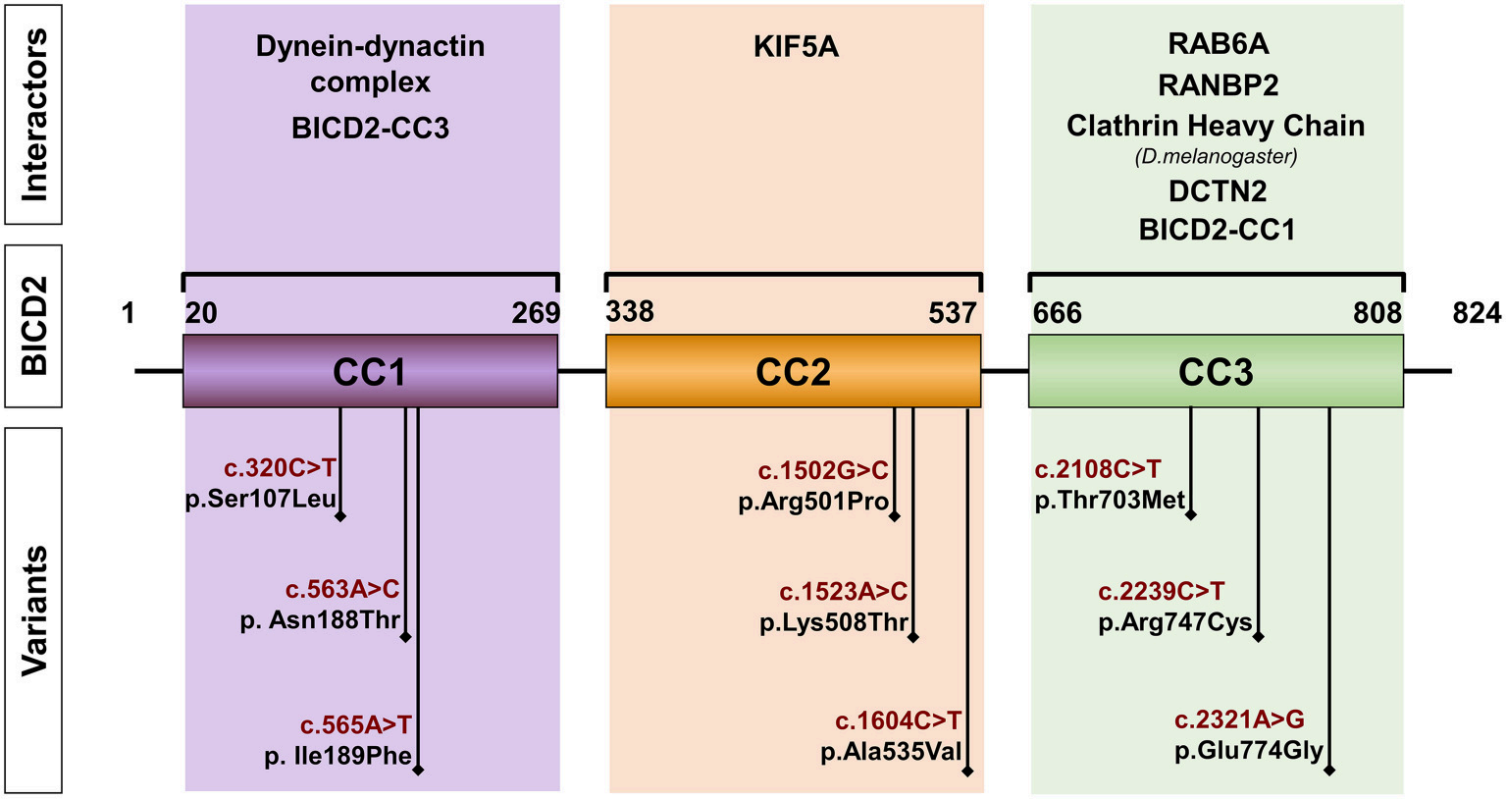
**Schematic representation of BICD2**. The diagram depicts the coiled-coil domains (CC) of BICD2 indicating the corresponding interacting partners. The position of variants, found in patients with SMALED2 is shown (Neveling et al., 2013; Oates et al., 2013; Peeters et al., 2013; Synofzik et al., 2014; Rossor et al., 2015).

*BICD2* localizes in chromosomal region 9q22.31 and encodes a canonical isoform of 824 amino acids. All the variants found in patients with SMALED2 are heterozygous and lead to single amino acid substitutions (Figure 1). The most common variant reported is the c.320C>T, p.Ser107Leu, found in 29 of 49 cases corresponding to 5 of 13 families described with SMALED2. This variant is located within a CpG dinucleotide (Neveling et al., 2013; Oates et al., 2013; Peeters et al., 2013; Bansagi et al., 2015; Rossor et al., 2015). The cytosine-guanine (CpG) dinucleotide is considered a hotspot for pathological variants. Cytosine is subject for methylation however, spontaneous deamination of 5-methylcytosine may occur, yielding thymine instead (Shen et al., 1994).

## BICD2 is highly conserved among species

**Figure 2:**
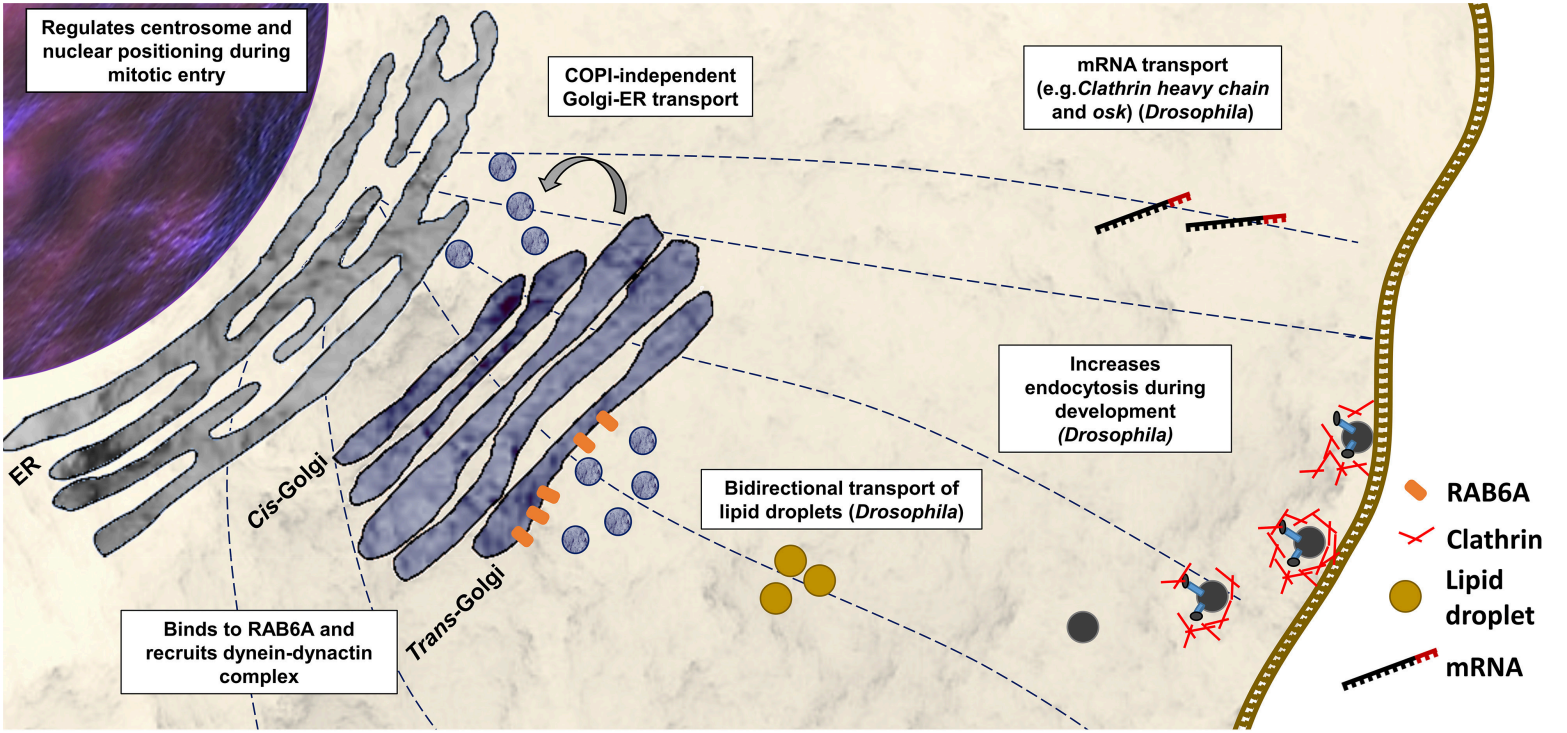
**Functions of BICD2 in the cell**. Cellular process with BICD2 involvement as described from studies in mammals or *Drosophila* are shown. BICD2 binds to RAB6A and recruits the dynein-dynactin complex to the trans-Golgi to facilitate COPI-independent Golgi-ER transport (Hoogenraad et al., 2001, 2003; Matanis et al., 2002). Prior mitosis (during G2 phase), BICD2 binds to a component of the nuclear pore complex, the RANBP2, and recruits dynein-dynactin to keep centrosomes tethered to the nucleus (Splinter et al., 2012). Studies in *D. melanogaster* have shown that BicD participates in mRNA transport (Bullock and Ish-Horowicz, 2001), endocytosis together with clathrin heavy chain (Vazquez-Pianzola et al., 2014), and in the regulation of bi-directional transport of lipid droplets (Larsen et al., 2008).

*Bicaudal-D (BicD)* was identified first in *Drosophila melanogaster* in a mutant screen for dominant maternal-effect proteins. Loss-of-function mutations in BicD interfere with the determination of the oocyte, while gain-of-function substitutions (e.g., p.Glu224Lys, p.Phe684Ile) disrupt the establishment of anterior and posterior polarity giving rise to bicaudal (two tails) embryos (Mohler and Wieschaus, 1986; Steward, 1987; Schüpbach and Wieschaus, 1991). *BicD* null mutations are recessively lethal (Ran et al., 1994). Further studies show that BicD is a component of dynein-based transport and is implicated in the transport of mRNA (e.g., *clathrin heavy chain* and *osk*) to specific cellular regions of the fly during oogenesis and embryogenesis (Figure 2; Bullock and Ish-Horowicz, 2001). Experimental evidence suggested that BicD, together with clathrin heavy chain, mediates endocytosis during oocyte development (Vazquez-Pianzola et al., 2014). BicD is also involved in the regulation of bi-directional transport of lipid droplets (Larsen et al., 2008).

There is only one *BicD* gene in invertebrates, while mammals have two homologs *BICD1* and *BICD2*. BICD1 and BICD2 interact with the dynein-dynactin motor complex (Hoogenraad et al., 2001), and both have been associated with Golgi-ER transport (Matanis et al., 2002). Studies in *Bicd2* knockout mice revealed, that BICD2 has an important role in neuronal cell migration (Figure 2) (Jaarsma et al., 2014). The *Bicd2* knockout mice present severe developmental defects of the cerebellar cortex, however other brain regions and spinal cord appear normal (Jaarsma et al., 2014).

## BICD2 structure and organization

Studies in *Drosophila* revealed that more than half of the BicD protein consists of heptad repeats. A heptad repeat is a repeating pattern of seven amino acids where hydrophobic residues are preferentially located at positions 1 and 4 (McLachlan and Karn, 1983). These heptad repeats mediate the packaging of one helix against another, forming coiled-coil (CC) structures made up of two or three protein molecules and resulting in homo- or heterodimers or in multimers (Bruccoleri et al., 1986). The BICD protein is predicted to consist of CC domains: the N-terminal domain contains the coiled-coil segment 1 (CC1) and the coiled-coil segment 2 (CC2), and the C-terminal contains the coiled-coil segment 3 (CC3). Studies in *Drosophila* and mammals suggested, that BICD interacts with itself forming homodimers (Oh et al., 2000; Hoogenraad et al., 2001). In the case of BICD2, the segment 1 of the N-terminal domain (CC1, amino acids 10–321) binds to the C-terminal segment (CC3, amino acids 706–810) (Hoogenraad et al., 2001). A hypothetical model proposes that as a result of this interaction between segments, the soluble free BICD2 folds up, and only when C-terminal segment engages in an interaction with other proteins, the N-terminal segment of the BICD2 becomes available for interaction with other proteins (Hoogenraad et al., 2001). These protein interactions seem to be determining for the cellular distribution of BICD2 and the fulfillment of its specific function (Figures 1, 2).

## The role of BICD2 in the golgi apparatus and dynein-dynactin complex

One of the most studied binding partners of BICD2 is the dynein-dynactin complex. Cytoplasmic dynein is the major responsible motor protein for transporting a large variety of cargos toward the minus ends of microtubules (Kardon and Vale, 2009). Dynactin is a protein complex that stimulates dynein processivity and participates in cargo binding (Holleran et al., 1998; Schroer, 2004). Dynein and dynactin directly bind to each other through the interaction of dynein intermediate chain and dynactin subunit p150 (Vaughan et al., 1995; Waterman-Storer et al., 1995; King et al., 2003). The N-terminal domain of BICD2 binds to the dynein-dynactin complex via interaction with the p50 subunit of the dynactin complex (Hoogenraad et al., 2001). Studies *in vivo* and *in vitro* suggested, that the N-terminus of BICD2 promotes a stable interaction between dynein and dynactin (Splinter et al., 2012). In this context it is particularly interesting to mention that mutations in cytoplasmic dynein DYNC1H1 causes SMALED1 (Harms et al., 2012; Tsurusaki et al., 2012; Peeters et al., 2015). See the Review Jaarsma and Hoogenraad (2015).

The C-terminal segment of BICD2 shows the highest degree of evolutionary conservation and is described as the cargo-binding domain (Hoogenraad et al., 2001; Terenzio and Schiavo, 2010). BICD2 is considered a linker protein that acts between a cargo bound to its C-terminal segment and the dynein-dynactin complex associated to its N-terminal segment. The C-terminal segment of BICD2 binds strongly to the active form of the RAB6A GTPase and is responsible for Golgi targeting (Hoogenraad et al., 2001, 2003).

RAB6A is part of a big RAB family of more than 60 members (Zerial and McBride, 2001). RAB proteins shift dynamically between an active membrane-associated GTP-bound form and an inactive cytosolic GDP-bound form (Pfeffer, 2001; Zerial and McBride, 2001). RAB proteins are associated with membranes via geranylgeranyl groups that are attached to cysteine residues at the C-terminus (Stenmark, 2009). Guanine nucleotide exchange factors (GEFs) are responsible for the activation of RAB proteins by catalyzing the exchange of GDP for GTP (Delprato et al., 2004; Stenmark, 2009). Experimental evidence suggested that GEFs are responsible for targeting the RAB proteins to specific membranes (Gerondopoulos et al., 2012; Blümer et al., 2013). The active, membrane-bound RAB proteins are involved in membrane traffic, by binding with specific proteins (Grosshans et al., 2006).

The small GTPase RAB6A is involved in intra-Golgi transport. Several studies support that RAB6A coordinates the retrograde COPI-independent Golgi-ER pathway, which is considered a recycling route for Golgi-resident glycosylation enzymes (Martinez et al., 1997; Girod et al., 1999; White et al., 1999; Storrie et al., 2000). The active form of RAB6A (GTP-bound) recruits BICD2 to the *trans*-Golgi membrane via direct interaction with the C-terminal domain of BICD2 (Hoogenraad et al., 2001; Matanis et al., 2002; Short et al., 2002; Bergbrede et al., 2009; Matsuto et al., 2015). Recent reports suggest that BICD2 may stabilize the active RAB6A, by inhibiting its GTPase activity and thus increasing the GTP-bound membrane-associated RAB6A (Matsuto et al., 2015). Once the C-terminal segment of BICD2 binds to RAB6A, the N-terminal segment of BICD2 becomes available and recruits the dynein-dynactin complex. This recruitment is considered a critical step for the microtubule retrograde traffic. In this manner, a coordinated action between RAB6A, BICD2 and the dynein-dynactin complex controls COPI-independent Golgi-ER transport (Matanis et al., 2002).

Hence BICD2 has an abundant CC structure, localizes at the Golgi complex, and interacts with a member of the RAB family of GTPases (RAB6A), it is considered to be a golgin (Barr and Short, 2003; Short et al., 2005; Goud and Gleeson, 2010).

Golgins are proteins associated with the Golgi apparatus and help to maintain the organized architecture of this dynamic organelle. One type of golgins consists of resident transmembrane-Golgi proteins which are necessary for tethering membranes together (Barr and Short, 2003). Another type of golgins corresponds to proteins that are recruited to the Golgi apparatus, and are associated with components of the trafficking machinery. BICD2 is included in this last class (Barr and Short, 2003).

## Mutations in BICD2 cause golgi fragmentation

The effects of the BICD2 domains on the integrity of the Golgi apparatus have been studied in detail (Hoogenraad et al., 2001, 2003; Splinter et al., 2012). Although the C-terminal domain targets the BICD2 to the Golgi apparatus, the overexpression of the N-terminal segment perturbs the Golgi organization (Hoogenraad et al., 2001). Fragmentation of the Golgi apparatus has been associated with inhibition of dynein function (Burkhardt et al., 1997; Harada et al., 1998; Quintyne et al., 1999).

Based on the association of BICD2 with RAB6A and Golgi targeting, we performed immunofluorescent staining of the Golgi apparatus of fibroblast cells derived from the patients carrying heterozygous mutations in *BICD2*.

**Figure 3:**
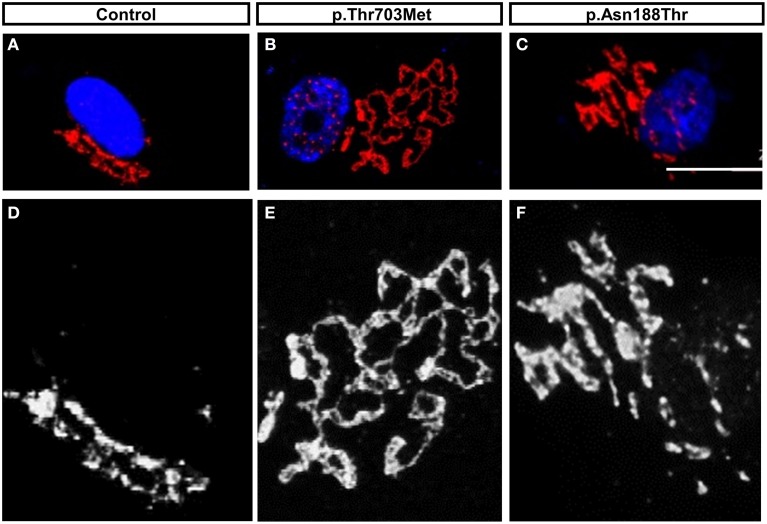
**The mutations in BICD2, p.Thr703Leu and p.Asn188Thr, triggered Golgi-fragmentation in Primary Fibroblast Cells**. **(A–C)** Modified data as published previously (Neveling et al., 2013), showing the Golgi apparatus of control fibroblasts and patients fibroblasts. The Golgi apparatus was immunostained with antibody against the Golgi marker ML160. **(D–F)** Magnification in monochrome of **(A–C)** for better visualization and comparison. Please note the prominent unfolded and disperse structure of the Golgi apparatus in cells carrying the mutation p.Thr703Leu and the intermedium or mild dispersion in the cells carrying the mutations p.Asn188Thr. For detail information and quantification, please read (Neveling et al., 2013).

We observed a prominent fragmentation of the Golgi apparatus in primary fibroblast cells harboring the p.Thr703Leu substitution (C-terminal) and a milder fragmentation in cells with the p.Asn188Thr substitution (N-terminal) (Neveling et al., 2013) (Figure 3). The individuals carrying the mutation p.Thr703Leu show a more severe phenotype including congenital contractures, while the individuals carrying the mutation p.Asn188Thr do not present contractures. Thus, in the case of these two cell lines showing Golgi fragmentation, the grade of fragmentation seems to correlate with the severity of the disease (Neveling et al., 2013). Although, Golgi fragmentation is not a common feature in all the cell lines carrying mutations in BICD2 (Oates et al., 2013; Peeters et al., 2013).

The BICD2 mutations p.Thr703Leu and p.Asn188Thr may cause fragmentation of the Golgi apparatus due to conformational changes of BICD2 that alter protein function and affect the structure of the Golgi. How amino acid substitution at the positions Thr703 and Asn188 impact intramolecular interactions and probably protein folding remains elusive, as there is no crystalloid structure of BICD2 or the region harboring these mutations available. The alteration in the Golgi structure could also be a secondary effect of alteration in the microtubule organization. The integrity and positioning of the Golgi apparatus seems to have an important role in neuronal polarization and development (de Anda et al., 2005).

## Potential consequences of golgi fragmentation on neuronal development and maintenance

Proper development of neurons is vital to fulfill and maintain the precise connectivity of the nervous system. Neurons are among the most polarized cell types and consist of a cell body from which specialized structures emerge, the neurites. In a very coordinated process, only one of the neurites grows long becoming an axon, while the other neurites acquire dendritic identity (Goslin et al., 1989; Craig and Banker, 1994; Polleux and Snider, 2010).

Previous studies have shown that in completely undifferentiated neurons, the centrosomes, Golgi apparatus and late/recycling endosomes cluster together to the area where the first neurite will form, which is in turn opposite from the plane of the last mitotic division (de Anda et al., 2005). The coordinated activity of these organelles is necessary for the polarization of the neuron.

Besides axon development, it has been suggested that the Golgi apparatus has an important role in dendrite maintenance and shape (Horton and Ehlers, 2003; Lewis and Polleux, 2012; Ori-McKenney et al., 2012). Non-neuronal cells contain one Golgi apparatus while many neurons contain several Golgi, the somatic Golgi and the dendritic Golgi centers called “Golgi outposts.” These dendritic Golgi outposts function similarly to the somatic Golgi, providing dendrites with substantial secretory capacity. The Golgi outposts appear during early neuronal differentiation, and may form as fragments derived from somatic Golgi that expand into dendrites, as the dendrites grow (Horton and Ehlers, 2003). The Golgi structure, dynamics and localization seem to have an important effect on neuronal development and maintenance, and an alteration in any of them could trigger degeneration or failure in correct maturation.

Additionally to SMALED2, other motor neurodegenerative diseases like ALS, have been associated with Golgi fragmentation (Gonatas et al., 1992; Mourelatos et al., 1994). However, it is still not clear why motor neurons but not other neuronal cells are affected. This effect might be attributable, to specific requirements of motor neurons during development that are different from other neuronal populations.

In conclusion, pathogenic substitutions in BICD2 causes SMALED2 with an autosomal dominant inheritance. Patients with SMALED2 show slowly to non-progressive muscle weakness of proximal and distal mainly of lower extremities, with a quite broad clinical spectrum. The majority of patients show congenital or early onset with foot deformities and joint contractures. However, some patients present a late onset and have very mild and non-progressive appearance. Commonly seems to be a wide aperture of the upper body (body-building shape). BICD2 is a golgin adaptor protein. The various BICD2 substitutions found in patients with SMALED2 impair interaction to other binding partners such as dynein-dynactin, RAB6A etc. and thus impair most likely cellular processes such as axonal transport, vesicular transport, Golgi integrity etc, which however needs further intensive functional studies. It is not understood why these mutations cause specifically loss of spinal motor neurons, since BICD2 is expressed in all neurons, is essential in neuronal migration and in positioning of the nucleus in the cell. There is also a quite broad phenotypic variability even in patients with the same mutation, suggesting the influence of modifying factors, which also need to be investigated.

## Conflict of interest statement

The authors declare that the research was conducted in the absence of any commercial or financial relationships that could be construed as a potential conflict of interest.
